# Growth kinetics of white graphene (h-BN) on a planarised Ni foil surface

**DOI:** 10.1038/srep11985

**Published:** 2015-07-09

**Authors:** Hyunjin Cho, Sungchan Park, Dong-Il Won, Sang Ook Kang, Seong-Soo Pyo, Dong-Ik Kim, Soo Min Kim, Hwan Chul Kim, Myung Jong Kim

**Affiliations:** 1 Soft Innovative Materials Research Center, Korea Institute of Science and Technology, Chudong-ro 92, Bongdong-eup, Wanju-gun, Jeollabuk-do 565-905, Republic of Korea; 2 Department of Advanced Materials Chemistry, Korea University, Sejong, Chungnam 339-700, Republic of Korea; 3 High Temp. Energy Materials Research Center, Korea Institute of Science and Technology, Hwarang-ro 14gil-5, Seongbuk-gu, Seoul 136-791, Republic of Korea; 4 Department of Organic Materials and Fiber Engineering, Chonbuk National University, 567, Baekje-daero, Deokjin-gu, Jeonju-si, Jeollabuk-do 561-756, Republic of Korea

## Abstract

The morphology of the surface and the grain orientation of metal catalysts have been considered to be two important factors for the growth of white graphene (h-BN) by chemical vapour deposition (CVD). We report a correlation between the growth rate of h-BN and the orientation of the nickel grains. The surface of the nickel (Ni) foil was first polished by electrochemical polishing (ECP) and subsequently annealed in hydrogen at atmospheric pressure to suppress the effect of the surface morphology. Atmospheric annealing with hydrogen reduced the nucleation sites of h-BN, which induced a large crystal size mainly grown from the grain boundary with few other nucleation sites in the Ni foil. A higher growth rate was observed from the Ni grains that had the {110} or {100} orientation due to their higher surface energy.

White graphene (hexagonal boron nitride, h-BN) is an analogue of graphene in which one carbon atom forms sp^2^ hybridised bonding with three other carbon atoms. Here, a boron atom forms sp^2^ hybridised bonding with three nitrogen atoms, and a nitrogen atom bonds with three boron atoms in the honeycomb structure of h-BN. This is considered to be the ideal substrate for a dielectric layer in various graphene electric devices due to its flat surface and high band gap (5.9 eV)^1^,^2^,^3^,^4^,^5^. Its other exceptional properties, such as superb oxidation resistance at 800 °C, excellent chemical resistance to acid, high thermal conductivity, and superior elastic modulus^6^,^7^,^8^,^9^,^10^, are also advantageous for a wide range of applications, especially for graphene electric devices^1^,^2^, ultraviolet-light emitters^3^,^4^,^5^, protective coating materials^11^, and composites^12^. These benefits and great uses have led to intense research on h-BN. Because the synthesis of new materials is a major part in the initial stage of research, various synthetic methods, such as exfoliation of h-BN flakes^13^,^14^, the chemical vapour deposition (CVD) method^15^,^16^,^17^,^18^,^19^,^20^, and a sputter method, have been attempted. In particular, the CVD method has been recently highlighted due to its capability to synthesise large area h-BN with high quality and controllable thickness^10^,^17^. Through this CVD method, h-BN was synthesised on the metal catalysts using molecular precursors, such as borazine (B_3_N_3_H_6_)^15^,^16^, ammonia borane (BH_3_NH_3_)^10^,^11^,^17^, and diborane (B_2_H_6_) with ammonia^20^. For metal catalysts, a variety of metal catalysts have been attempted in the form of foils (Cu foil^10^,^11^,^17^,^20^,^21^,^22^, Ni foil^15^,^16^,^18^,^23^,^24^, and Pt foil^25^,^26^) and metal thin films^27^,^28^,^29^,^30^,^31^ by deposition. Among those, foils are preferred to metal thin films due to their price competitiveness and the advantage of a large areal synthesis.

The growth of h-BN on metal catalysts is substantially influenced by the interaction between the metal catalysts and precursors^32^. Thus, the surface properties of the metal catalysts, such as surface adsorption^33^ and catalytic activity to dehydrogenate precursors^34^,^35^, depend on surface morphology and crystalline orientation^21^,^23^ and should be considered to elucidate a growth mechanism. There are a few studies on the effect of crystal orientation and surface morphology of metal catalysts as foils^36^, as well as thin films, for h-BN growth^17^,^21^,^22^,^23^,^24^. Regarding metal foils, Guo *et al.* recently reported triangular h-BN domain formation on the {100} plane of annealed Cu foil^21^, and Lee *et al.* also reported that Ni {100} was more preferred than Ni {111} for the h-BN growth^23^. Their research, however, was limited because the morphology effect of metal foil is still involved in the h-BN growth experiment.

Here, we report the detailed correlation between the growth rate of h-BN and grain orientation of a Ni polycrystalline foil. The surface morphology was controlled by an ECP method and subsequent hydrogen atmosphere annealing at low or atmospheric pressure (ECP/LPH_2_ and ECP/APH_2_). In particular, research related to growth of h-BN and ECP/APH_2_ on Ni surface has not yet reported. We, therefore, found that the number of nucleations and domain shape of h-BN were clearly distinguished depending on the annealing pressure. ECP/APH_2_ reduced microstructural heterogeneous sites in the Ni foil, which induces h-BN nucleation. Thus, we can obtain large domain sized h-BN mainly grown from the grain boundary with few other nucleation sites in the Ni foil. Because this method suppressed the effect of the surface morphology, the relationship between the growth rate of h-BN and the grain orientation of metal catalyst has been obviously clarified. The growth rate of h-BN was faster at the Ni {110} or {100} but slower at Ni of {111} because each grain has the different sticking coefficient (S) for BN radicals and BN molecules containing boron, nitrogen and hydrogen, reflecting the surface energy of each grain.

## Results

Even though Ni foils have been used as catalysts for h-BN synthesis due to the advantage of large areal synthesis compared to Ni thin films, they have the disadvantage that their surface morphology and purity depend on the manufacturers. Ni foils were purchased from the Nilaco Corporation for our experiments.

**Figure 1:**
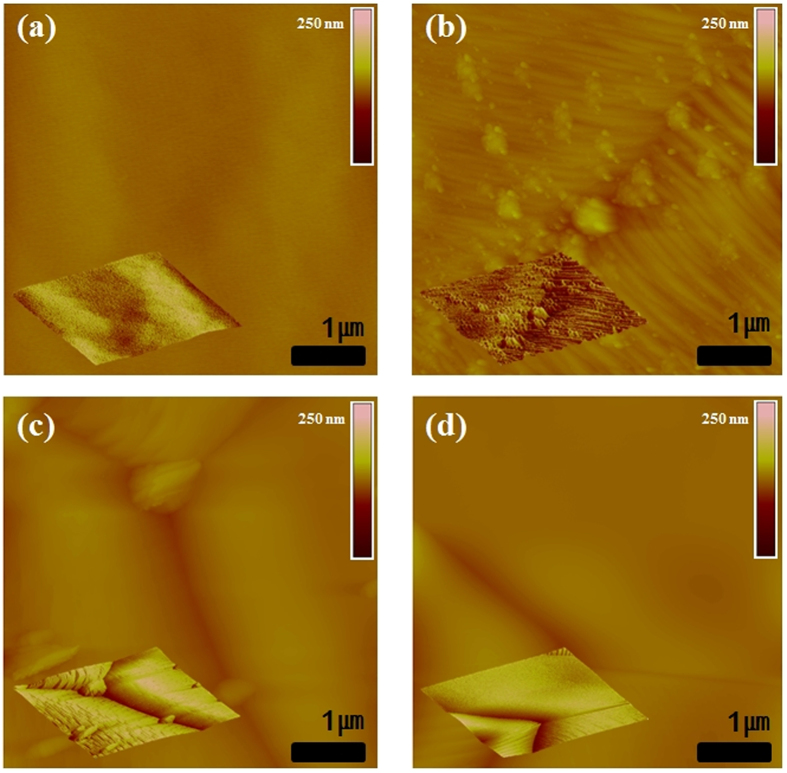
The comparison AFM images of (**a**) the raw Ni foil, (**b**) the Ni foil with LPH_2_, (**c**) the Ni foil with ECP/LPH_2_ and (**d**) the Ni foil with ECP/APH_2_.

**Figure 2:**
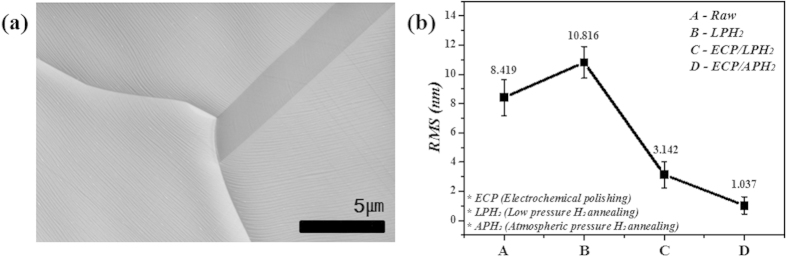
(**a**) A SEM image of the Ni foil with ECP/APH_2_ and (**b**) the RMS comparison of the Ni foil samples measured by AFM: A-the raw Ni foil, B- the Ni foil with LPH_2_, C-the Ni foil with ECP/LPH_2_, D-the Ni foil with ECP/APH_2_.

H_2_ annealing of the Ni foil is a generic procedure performed right before the growth procedure, as H_2_ annealing can provide large grains and clean surfaces for uniform h-BN growth. A variety of particles (Ni, SiO_2_, Al_2_O_3_, Cr, Co, Mn, and MgO), however, were formed on the surface after low pressure (LP) H_2_ annealing (200 mTorr), as the Ni foils have metal impurities and residual of Si lubricant coatings on the surface from the rolling process. The chemical elements of the particles were analysed by energy-dispersive X-ray spectroscopy (EDS) in a scanning electron microscope (SEM) and are presented in Supplementary Fig. S2. From the viewpoint of growth mechanism, various particles and poor surface condition of catalyst can easily act as nucleation sites for the growth. Thus, in order to remove the particles, including residual of Si lubricants, and to planarise the surface that has edges, steps and uneven surface, ECP process was attempted, and then LP H_2_ annealing was followed. As shown in Fig. 1(c) and Fig. 2(b), the number of particles and the surface roughness substantially decreased. The root mean square (RMS) was evaluated by using the atomic force microscopy (AFM) (Fig. 1). Among all conditions, the complete removal of particles and lowest surface roughness of Ni foil were acquired by ECP/APH_2_ as shown in the SEM images (Fig. 2(a,b)). The RMS value of the condition was 1.037 nm with a small deviation. In our results, the ECP/AP process provided the platform to clean and planarise the Ni foil on the nanometre scale. Detailed discussion about the hydrogen annealing under atmospheric pressure (APH_2_) will be addressed in the next section and we provided new abbreviations about the detailed experimental conditions in supplementary information.

**Figure 3:**
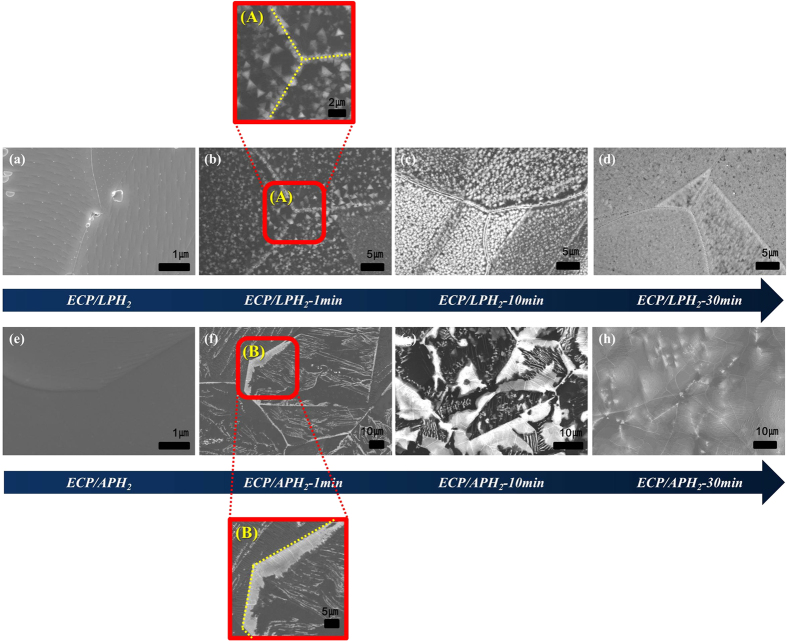
Time evolution of h-BN growth morphology on the Ni foils with ECP/LPH_2_ and ECP/APH_2_: (**a**,**e**) are SEM images of as annealed sample. (**b**,**f**), (**c**,**g**), and (**d**,**h**) are SEM images of h-BN growth for 1 min, 10 min, and 30 min respectively. The first row and the second row are the Ni foil with ECP/LPH_2_ and ECP/APH_2_, respectively. (**A**) and (**B**) are magnified SEM images taken from the area of Figure (**b**) and Figure (**f**) respectively.

**Figure 4:**
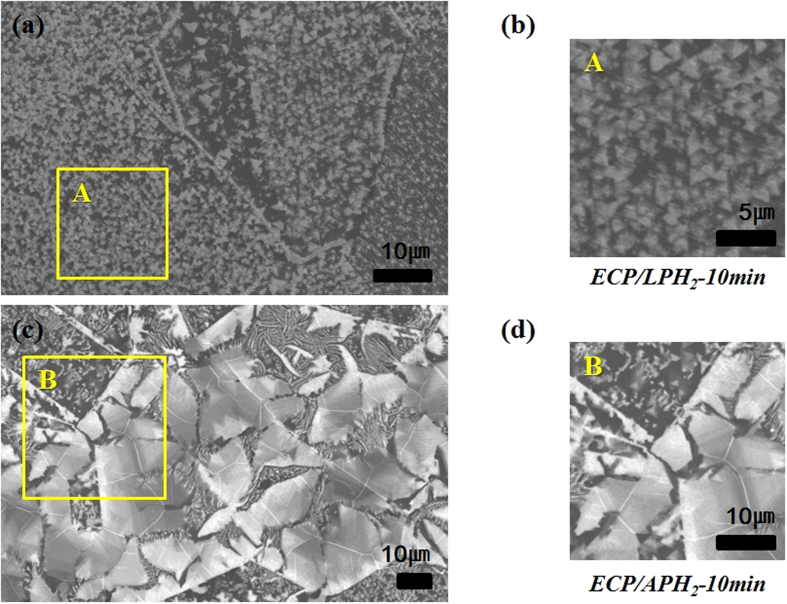
The comparison of SEM images of the Ni foil with ECP/LPH_2_-10 min (**a,b**) and ECP/APH_2_-10 min (**c,d**).

We assumed that the growth feature of h-BN would be affected by the surface roughness of the Ni foil, similar to graphene growth^37^. The Ni foils went through either ECP/LPH_2_ or ECP/APH_2_ before the growth procedure in order to understand how the surface morphology of the Ni surface affects h-BN growth. Dramatic contrast in the h-BN growth kinetics was found, as shown in Figs 3 and 4. While dense triangular domains were observed on the Ni foil with ECP/LPH_2_ (Fig. 4(a,b)), large area and continuous domains were observed on the Ni foil with ECP/APH_2_ (Fig. 4(c,d)). Due to the higher surface roughness, small size h-BN domains were formed from the numerous nucleation sites, and thus, distinctive kinetics depending on the crystalline orientation of Ni were not observed in ECP/LPH_2_ condition. In the case of ECP/APH_2_, nucleation sites were limited to the grain boundary with scarce nucleation on the surface of the grains, such that the growth kinetics of h-BN was exclusively affected by the orientation of the Ni substrate. Detailed discussion about the growth kinetics will be addressed in the next section.

## Discussion

Time evolution data of the h-BN growth was indicated in Fig. 3 in order to obviously describe kinetics of h-BN growth. The SEM images in Fig. 3(a,e) indicated the surface morphology of ECP/LPH_2_ and ECP/APH_2_. In particular, the particles and defects sites such as groves and wrinkles were existed on the surface of the Ni foil with ECP/LPH_2_. In contrast, all particles were clearly eliminated and the defect sites were reduced due to the effect of ECP/APH_2_.

Similar to ECP process, H_2_ annealing under atmospheric pressure (APH_2_) is able to form flat surfaces by annihilating native oxide and contaminations on the surface and by rearranging the unstable surface defect sites such as dislocations, wrinkles, pinning at surface, grooves, and stepped terraces^36^,^37^,^38^,^39^,^40^,^41^,^42^,^43^.

In particular, the high impingement rate of hydrogen molecules to the Ni surface might play an important role in planarizing Ni surface by reducing NiO and thus enhancing surface mobility of Ni atoms^40^,^41^,^42^ Considering that the melting point of solid NiO (1955 ^o^C) is higher that of pure solid nickel Ni (1455 ^o^C), the surface mobility of nickel atoms in solid NiO is presumed to be lower than that of Ni atoms in pure solid Ni. We believe that ECP/APH_2_ effectively reduced NiO into Ni, and thus surface Ni atoms rearranged with high surface mobility, resulting in planarised surface. Clear examination of oxidative state with surface chemistry level (*In-situ* XPS) needs to be done, but we leave it as a future work.

In order to understand the correlation between the surface roughness of Ni and the growth rate of h-BN, we roughly estimated the nucleation density and the growth rate of h-BN grown on two different types of the Ni foils (ECP/LPH_2_ and ECP/APH_2_) from the SEM images in Fig. 4(b,d). The surface roughness was indicated by RMS values in Fig. 2(b). While the nucleation density of h-BN increased with the surface roughness of Ni foils, the growth rate of h-BN has decreased with the increasing surface roughness of Ni foils as indicated in Supplementary Table S1.

Figure 3(b,f) indicated the SEM images of h-BN growth for 1 min on ECP/LPH_2_ and ECP/APH_2_ treated Ni foils respectively. Fig. 3(A,B) showed the magnified SEM image of h-BN growth taken from the grain boundary area of Fig. 3(b,f), indicating that the grain boundary of Ni foil became nucleation sites of h-BN growth because of higher surface energy than others. The yellow lines in Fig. 3(A,B) were used to describe the grain boundaries of Ni foil. Thus, The ECP/APH_2_ was significant process for the large-wide area of h-BN growth.

Furthermore, SEM data in Fig. 3(h) showed the growth feature at 30 min. Since Ni surface was fully covered irrespective of crystal orientations at 30 min (i.e. at equilibrium), the contrast of h-BN coverage on different crystal orientations at 10 min (ECP/APH_2_-10 min) is consequence of kinetics (i.e. different growth rate) rather than thermodynamics (i.e. equilibrium preference).

As shown in Fig. 4(a,c), we observed the higher growth rate of h-BN on the planarised Ni foils with ECP/APH_2_ compared to ECP/APH_2_ and elucidated the reasons below. In case of Cu foils, the nucleation of h-BN was hindered in smooth Cu surface due to increase in Gibbs energy barrier for nucleation^44^. We also observed low nucleation density of h-BN grown on the planarised Ni foils with ECP/APH_2_ in Figs 3 and 4 due to the same reason. When the nucleation density is higher, the coverage of h-BN on Ni becomes higher in the middle of the growth. Thus, the exposed catalytic area where BN radical and BN molecules are discomposed suddenly decreased and BN radical and BN molecules cannot be efficiently supplied to the edge of h-BN. As a result, the growth rate of h-BN slowed down on the rougher surface of Ni foil with ECP/LPH_2_ as shown in Fig. 4(a,b). Similar to what is reported on h-BN growth using Cu foils^44^, BN radical and BN molecules can be trapped in the defect sites such as particles, wrinkle, and grain boundaries^44^. The planarised surface of the Ni foil with ECP/APH_2_ could induce the substantial enhancement of the mobility of the BN radicals and BN molecules to have longer diffusion length by minimizing trap sites^44^. As a result, h-BN on the planarised Ni surface was large and wider area shown in Fig. 4(c,d), and the contrast of the h-BN growth rate on different crystal orientations became obvious due to the kinetics shift from surface diffusion limited to chemisorption limited.

**Figure 5:**
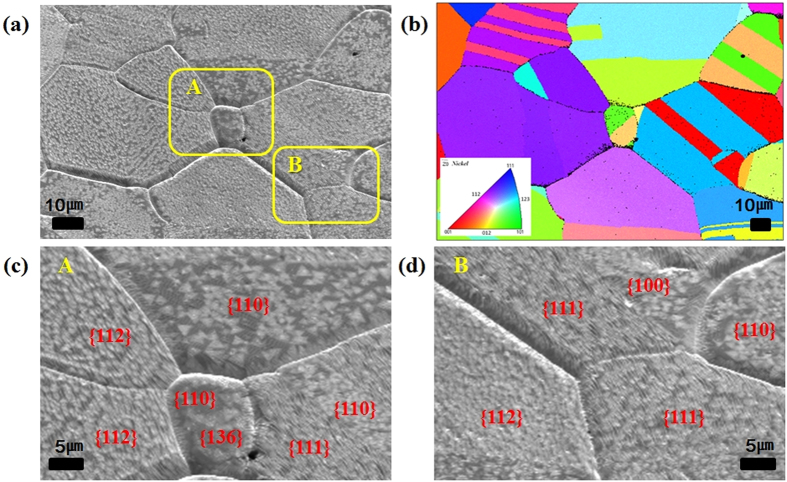
SEM images and EBSD analysis data of the Ni foil with ECP/LPH_2_-10 min: (**a**) SEM image, (**b**) EBSD orientation map, (**c**) SEM image of area A, and (**d**) SEM image of area B.

In order to gain an insight into the growth kinetics of h-BN in relation to the crystalline orientations, we performed Electron Back Scatter Diffraction (EBSD) analysis on the Ni foil where h-BN was grown after ECP/LPH_2_-10 min or ECP/APH_2_-10 min. The effect of ECP/LPH_2_-10 min is presented in the SEM image with the grain orientations (Fig. 5). They showed numerous triangular domains with small grain size, and the number of nucleations was similar in all crystalline orientations of the Ni foil. Only the triangular shape of h-BN was clearly shown on the Ni grains where the crystalline orientation was {100} or {110}, as shown in Fig. 5(c,d). We can assume that these triangular domains are nitrogen terminated, as boron-terminated domains have higher edge energy than nitrogen-terminated ones. Boron can easily react with a hydrogen atom on the surface of the {100} or {111} crystalline orientation to produce BHx gas^17^,^31^,^45^.

The domain size that represents the growth rate of h-BN made no difference. Growth of h-BN was affected by the surface conditions rather than by grain orientations, as shown in Fig. 5(c,d), and thus, the effect of gain orientation on the growth kinetics was not clarified.

**Figure 6:**
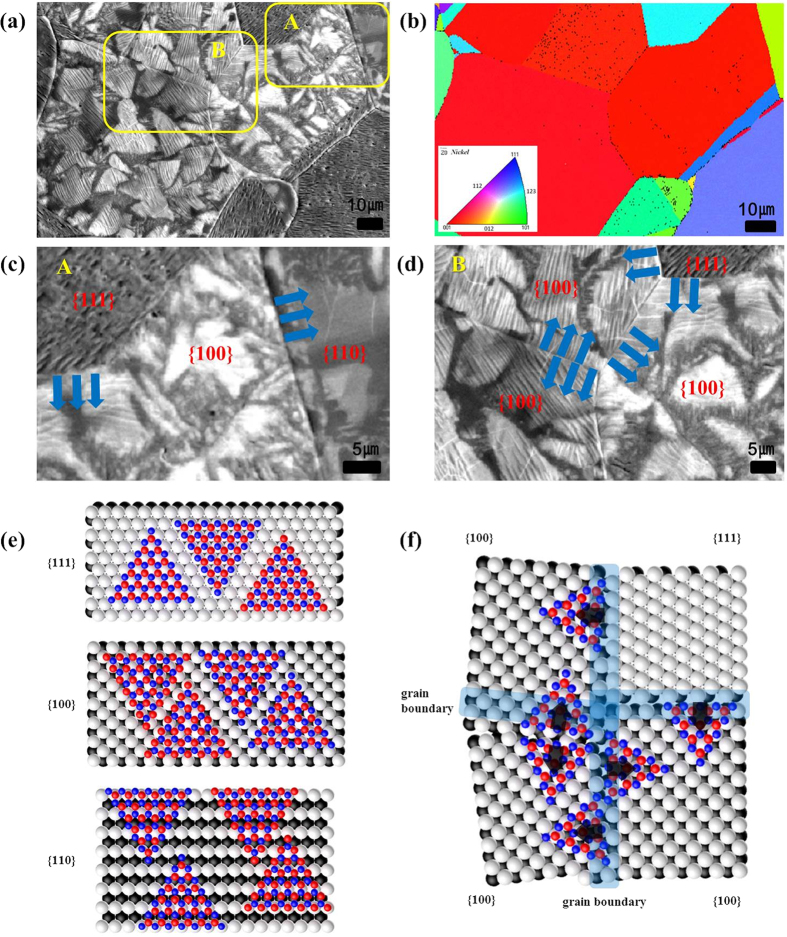
SEM images, scaled schematic models, and EBSD analysis data collected from h-BN grown on the Ni foil with ECP/APH_2_-10 min: (**a**) SEM image, (**b**) EBSD data, (**c**) SEM image of area A, and (**d**) SEM image of area B, (**e**) a scaled schematic model of h-BN growth on Ni {111}, {100}, and {110}, and (**f**) a scaled schematic model of the SEM image (**d**). Boron and nitrogen atoms are presented in red and blue respectively.

On the other hand, the shapes of h-BN grown on the Ni foil with ECP/APH_2_-10 min are shown in Fig. 6, and they were clearly distinguished from the results of the Ni foil with ECP/LPH_2_-10 min. Large area domains of h-BN were observed, and the nucleation density was low. The shape of domains appeared to be merged triangular domains. The growth of h-BN often initiated from grain boundaries and then continuously grew toward the grain centres, as presented in Fig. 6(d). The growth rate of h-BN observed in Fig. 6(c) showed contrast among 3 grains with different orientations. A higher growth rate of h-BN was found in the Ni {110} and Ni {100} than in the Ni {111}. Scaled schematic models of the h-BN growth on Ni are presented in Fig. 6(e,f) based on the recent research data on the lattice constants of h-BN and the Ni atom^46^. Though the lattice constant is well matched to the Ni {111} with 1.1% mismatch (Fig. 6(e)), the slowest growth rate was observed on the Ni {111}, implying that h-BN is not epitaxially grown from Ni crystals. Fig. 6(f) visualized the growth feature shown in the SEM image (Fig. 6(d)) in atomic scale, and this clarified our observation.

Another example that showed the contrasting growth rate dependence on the crystalline orientation is presented in Supplementary Fig. S5. We also found that the preferred crystalline orientations of Ni foils for h-BN growth were the {110}, {100} and {111} planes, in that order. The h-BN growth was barely observed in the {111} plane of the Ni foils in a given growth time.

In order to clearly identify the h-BN growth on Ni {111}, we have carried out the Auger Electron Spectroscopy (AES). As shown in Supplementary Fig. S6, the peaks of boron and nitrogen on Ni {111} by AES are not observed. This data indicated that h-BN was not grown on Ni {111} in contrast with Ni {110} and {100}. The reason of the high carbon peak is due to deposit carbon contamination by SEM, EBSD and AES during the intensive analysis and observation in the same area of Ni foil.

The difference in the growth rate of h-BN on the different orientations of the Ni grains can be understood from a kinetics point of view. The growth steps of h-BN consist of dissociative chemisorption of borazine on the Ni surface, polymerisation via dehydrogenation, and h-BN crystallisation.

As borazine (B_3_N_3_H_6_) transforms into a naphthalene-like structure (B_5_N_5_H_8_) or a diphenyl-like structure (B_6_N_6_H_10_) in gas phase at elevated temperature^47^,^48^,^49^,^50^,^51^, it can initially form polyborazylene by crosslinking via dehydrogenation and followed by polymerization on the catalytic substrate at elevated temperature. This polymeric species will be turned into h-BN, which is hexagonal boron nitride by annealing effect. Because Ni is an effective catalyst for the dehydrogenation of borazine and ammonia borane^52^,^53^, the polymerisation reaction via dehydrogenation will be fast, and thus it is not going to be the rate-determining step at the high growth temperature (1100 °C).

The rate-determining step will be the dissociative chemisorption of radicals and molecules containing boron, nitrogen and hydrogen which are constantly adsorbed and desorbed at high temperatures on the surface of the Ni foil. The adsorption rate (Rads) is proportional to the sticking coefficient (*S*) and flux (Hertz-Knudsen). Because flux was fixed in our experiments, the sticking coefficient is the key to elucidate the rate difference. Beebe *et al.* and Schouten *et al.* reported that the sticking coefficient (*S*) of methane (CH_4_) onto Ni grains decreases with {110}, {100} and {111}, in that order^45^,^54^. Thus, we can assume that the sticking coefficient (S) of radicals and molecules containing boron, nitrogen and hydrogen has the same order on Ni grains, as the sticking coefficient is predominately governed by the surface energy of the Ni grains. Vitos *et al.* reported that the surface energy depends on the crystalline orientation of Ni, and the surface energy of the Ni grains decreases with {110}, {100}, and {111} in that order^55^. This order reflects the contrast in the growth rate of h-BN.

**Figure 7:**
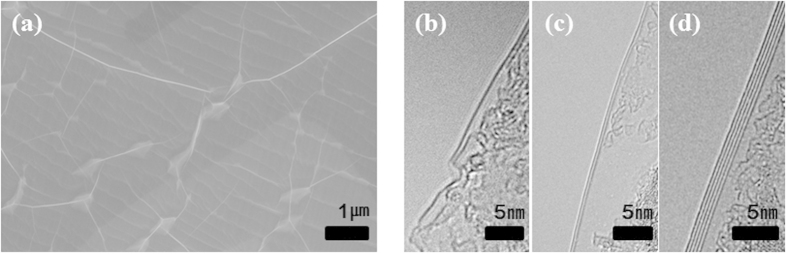
SEM image of full-covered h-BN on the Ni foil with ECP/APH_2_-30 min; (**b**–**d**) TEM image of h-BN.

The SEM image in Fig. 7(a) shows that h-BN has fully covered on the Ni foil with ECP/APH_2_-30 min. Granting that the foil was fully covered, h-BN has a thickness difference (i.e., difference in the number of layers) as shown in Fig. 7(b). The difference in growth rate depending on the crystalline orientation might lead to a difference in thickness that might be inevitable in h-BN growth on Ni foils.

In conclusion, the surface morphology and grain orientation are the key factors that govern the CVD growth of h-BN. In order to remove abundant nucleation sites on the Ni foil, ECP/APH_2_ were carried out. As a result, the surface of the Ni foil was clean and smooth on the nanometre scale, and thus, the growth of large area h-BN preferentially initiated from the grain boundary and spread over the Ni surfaces. A distinctive contrast in the growth rate on different grains was observed. The growth rate of h-BN of the Ni foil with ECP/APH_2_-10 min decreased with {110}, {100}, and {111}, in that order, as each grain has the different sticking coefficient (S) for radicals and molecules containing boron, nitrogen and hydrogen, reflecting the surface energy of each grain.

## Methods

White graphene (h-BN) was grown on the polycrystalline Ni foils (Nilaco Corporation) through the CVD method in Supplementary Fig. S1(a). Borazine (B_3_N_3_H_6_), synthesised from Kang’s group in Korea University in Supplementary Fig. S1(b), was used as a precursor^16^,^34^. In order to planarise the surface of the Ni foils, an ECP method (10 min) in conjunction with H_2_ annealing at LP or AP was attempted at 1100 °C. Then, a borazine and hydrogen mixture (approximately 6 mTorr) was introduced into the quartz tube furnace at 1100 °C for 1 min ~30 min to grow the h-BN. The sample was rapidly cooled to room temperature under a hydrogen atmosphere. For the characterisation of the h-BN, the grown film was transferred in a way similar to the CVD graphene transfer in Supplementary Fig. S1(c)^56^. The detailed synthesis and transfer method are explained in the supplementary information. We analysed two types of as-grown samples that went through H_2_ annealing under LP and AP. The crystalline orientations of the Ni grains were analysed by the EBSD, and other characterisations are summarised in Supplementary Fig. S3 and S4.

## Additional Information

**How to cite this article**: Cho, H. *et al.* Growth kinetics of white graphene (h-BN) on a planarised Ni foil surface. *Sci. Rep.* **5**, 11985; doi: 10.1038/srep11985 (2015).

## Supplementary Material

Supplementary Information
