# Supplementary material for: Growth kinetics of white graphene (h-BN) on a planarised Ni foil surface
Source: Sci Rep. 2015 Jul 9;5:11985. doi: 10.1038/srep11985 (PMC4496663; doi:10.1038/srep11985)
Supplement: Supplementary Information [file srep11985-s1.pdf]

Supplementary Information

Growth kinetics of white graphene (h-BN) on a planarised Ni foil surface

Hyunjin Cho[1,4], Sungchan Park[1], Dong-Il Won[2], Sang Ook Kang[2], Seong-Soo Pyo[3], Dong-Ik Kim[3], Soo Min Kim[1], Hwan Chul Kim[4] and Myung Jong Kim[1]*

[1]Soft Innovative Materials Research Center, Korea Institute of Science and Technology, Chudong-ro 92, Bongdong-eup, Wanju-gun, Jeollabuk-do 565-905, Republic of Korea

[2]Department of Advanced Materials Chemistry, Korea University, Sejong, Chungnam 339-700, Republic of Korea

[3]High Temp. Energy Materials Research Center, Korea Institute of Science and Technology, Hwarang-ro 14gil-5, Seongbuk-gu, Seoul 136-791, Republic of Korea

[4]Department of Organic Materials and Fiber Engineering, Chonbuk National University, 567, Baekjedaero, Deokjin-gu, Jeonju-si, Jeollabuk-do, 561-756, Republic of Korea

Content List:

1) Details for h-BN growth

2) SEM and EDS analyses of the Ni foil with LP

3) Raman, FT-IR, and XPS analyses of h-BN

4) TEM, SAED, and EELS analyses of h-BN

5) EBSD analysis of h-BN on the Ni foil with ECP/APH$_2$-10min

6) The analysis of h-BN growth in various grain boundaries by using (a) EBSD, (b) SEM and (c) AES of ECP/APH$_2$-10min.

7) The comparison among RMS value, nucleation density and growth rate.

We provided new abbreviations about the detailed experimental conditions.

1. The pristine Ni foil under low pressure annealing (The Ni foil with LP)

2. The nickel foil treated by electrochemical polishing (The Ni foil with ECP)

3. The ECP Ni foil under H_2 low pressure annealing (The Ni foil with ECP/LPH_2)

4. The ECP Ni foil under H_2 atmospheric pressure annealing (The Ni foil with ECP/APH_2)

5. The Ni foil with ECP/LP-H_2 for 1 min of h-BN growth (The Ni foil with ECP/LPH_2-1min)

6. The Ni foil with ECP/LP-H_2 for 10 min of h-BN growth (The Ni foil with ECP/LPH_2-10min)

7. The Ni foil with ECP/LP-H_2 for 30 min of h-BN growth (The Ni foil with ECP/LPH_2-30min)

8. The Ni foil with ECP/AP-H_2 for 1 min of h-BN growth (The Ni foil with ECP/APH_2-1min)

9. The Ni foil with ECP/AP-H_2 for 10 min of h-BN growth (The Ni foil with ECP/APH_2-10min)

10. The Ni foil with ECP/AP-H_2 for 30 min of h-BN growth (The Ni foil with ECP/APH_2-30min)

1) Details for h-BN growth

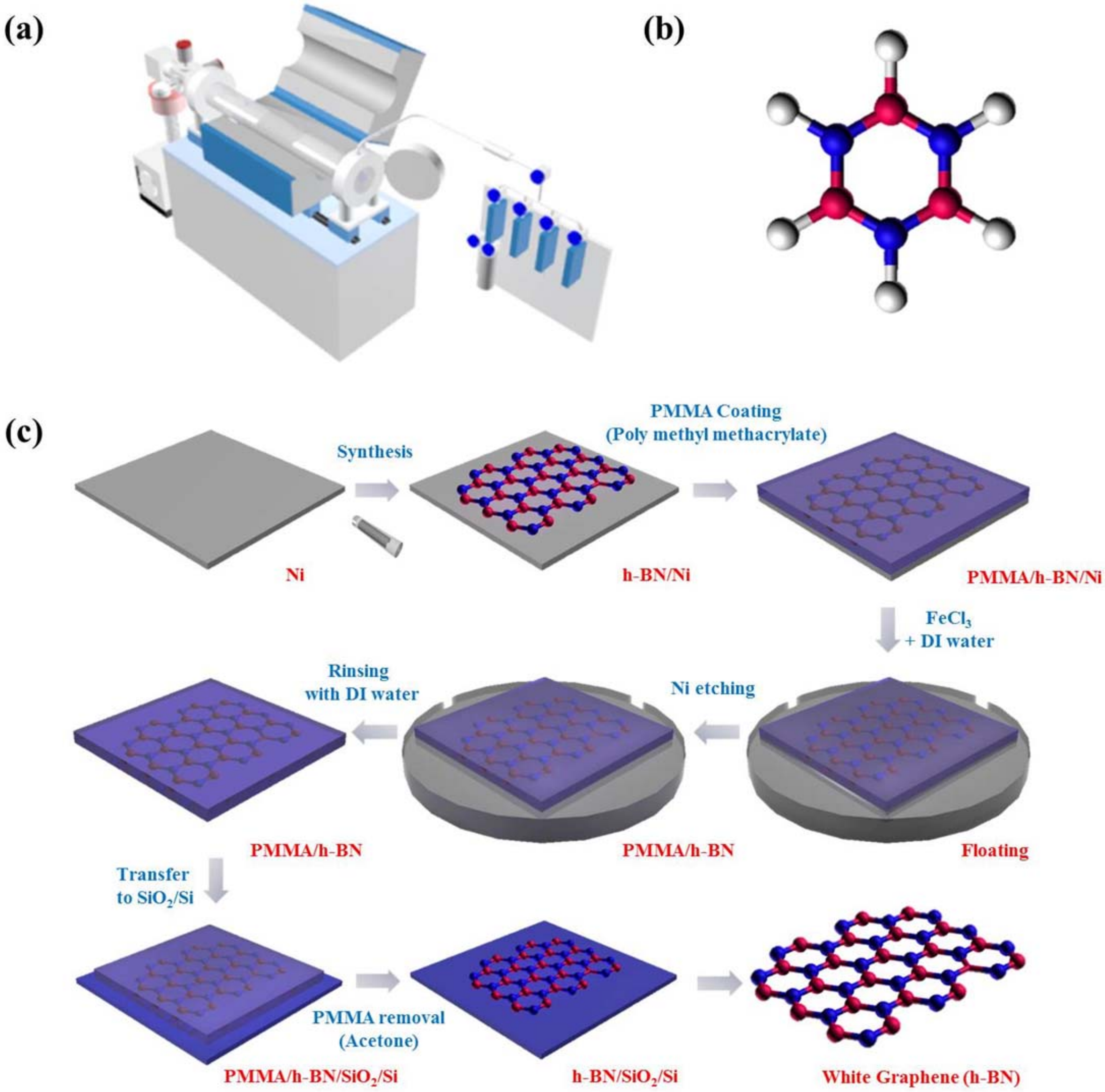

Figure S1. The CVD setup, precursors and transfer method of h-BN: (a) CVD setup, (b) Borazine ($B_3N_3H_6$), (c) Transfer methods.

Polycrystalline Ni foils (Nilaco Corporation) were used as the metal catalyst for the chemical vapour deposition (CVD) setup in Supplementary Fig. S1(a). Borazine ($B_3N_3H_6$) in Supplementary Fig. S1(b) was utilised as the precursor for preparing h-BN and was kept at -10°C in a canister with a

bubbler system in a chiller. Before growing the h-BN, the polycrystalline Ni foils were electrochemically polished in solution with a mixture of phosphoric acid and water for 10 min and then placed at the centre position of a quartz tube in the CVD. The annealing process under hydrogen (H_2) gas at low pressure (LP) or at atmospheric pressure (AP) was attempted at 1100°C for 30 min in order to control the morphology. After the annealing process, the base vacuum condition for growing h-BN was fixed under high vacuum pressure with a turbo molecular pump in order to improve the layer control. Borazine and hydrogen, subsequently, were introduced into the quartz tube at 1100°C and an approximate pressure of 6 mTorr for 1~30 min to grow the h-BN. Then, the Ni foils were rapidly cooled down to room temperature under a hydrogen atmosphere. For the characterisation analysis of the grown h-BN, the as-grown h-BN film was transferred onto various substrates by a method similar to graphene transfer as shown in Supplementary Fig. S1 (c)[1]; polymethyl methacrylate (PMMA) was spun onto the h-BN/Ni foil at 4200 rpm for 50 seconds, followed by the etching of the Ni foil using a Ni etchant (Iron(III) Chloride, $FeCl_3$). Transmission electron microscope (TEM) sample was transferred onto lacey carbon TEM grids to analyse the Electron Energy Loss Spectroscopy (EELS) and Selected Area Electron Diffraction (SAED) pattern. Additionally, we prepared two as-grown samples and carried out electron backscatter diffraction (EBSD, Bruker, FEI-Helios Nanolab 650) analysis for a detailed comparison of the different annealing methods. For other additional analyses of the h-BN, we employed many analysis apparatuses and techniques, including scanning electron microscope (SEM, FEI-Helios Nanolab 650), Energy-dispersive X-ray spectroscopy (EDS, EDAX), Raman spectroscopy (Horiba, LabRAM HR-UV-visible-NIR), X-Ray Photoelectron Spectroscopy (XPS, K-Alpha, Thermo Scientific), Fourier Transform Infrared Spectroscopy (FT-IR, Nicolet iS10), Auger Electron Spectroscopy (AES, Thermo Electron Corporation, Microlab 350) and TEM (FEI, Tecnai G2 F20, 200 kV).

2) SEM and EDS analysis of the Ni foil with LP.

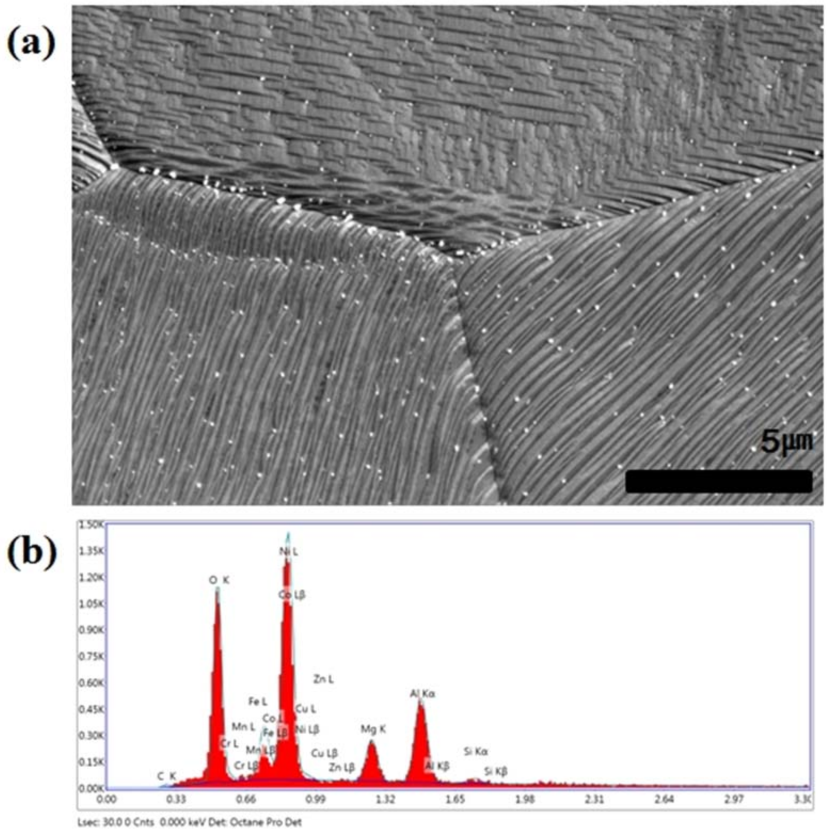

Figure S2. A SEM image (a) and EDS spectrum (b) acquired from the Ni foil with LP.

3) Raman, FT-IR, XPS analysis of h-BN

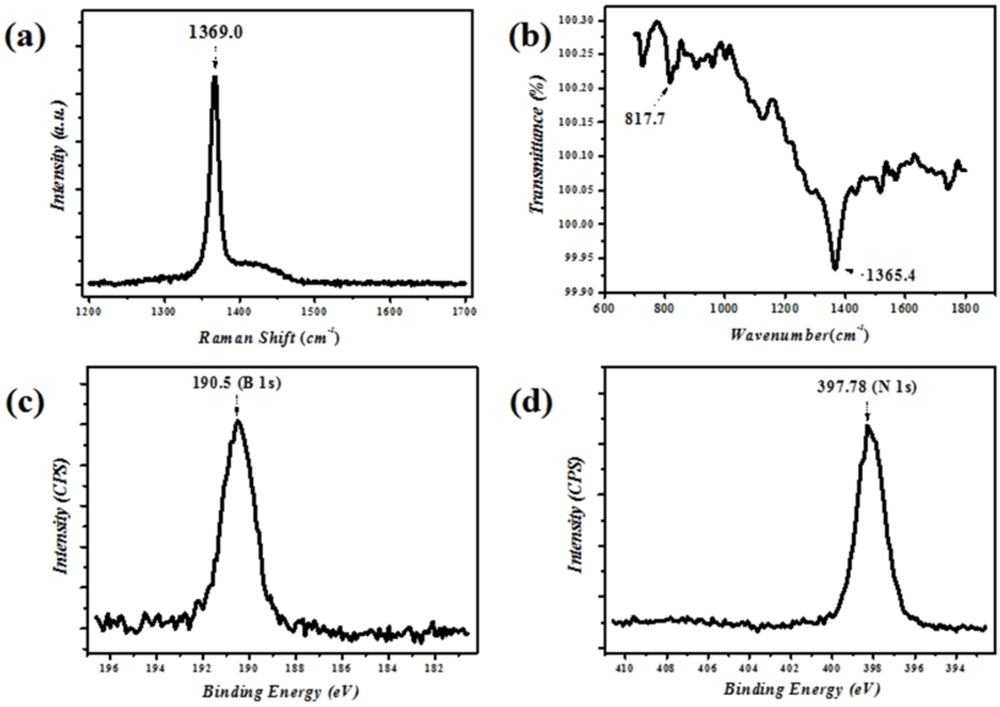

Figure S3. The characterisation of h-BN by various analysis methods; (a) Raman data of h-BN on a SiO_2/Si substrate, (b) FT-IR data of h-BN on a SiO_2/Si substrate, XPS data of h-BN on a SiO_2/Si substrate, (c) B 1s XPS spectrum, (d) N 1s XPS spectrum

In order to carry out the characterisation of the h-BN, various methods, such as Raman, FT-IR, XPS, TEM, SAED, and EELS, were employed. Supplementary Fig. S3 (a) shows the Raman data of the h-BN. Typically, the striking peak appears due to the E2g mode (B-N vibration) stretching, and it is similar to the G peak in carbon materials, such as graphene, carbon nanotubes and graphite.[2] In our experiments, a 514 nm laser was used, and the peak position was at 1369.0 cm^{-1}. It corresponded to the range of Raman data of h-BN. Furthermore, the full with half maximum (FWHM) value of the Raman data was mainly considered as the important factor for verifying the crystallinity of the h-BN. The FWHM value of our experiment was found to be 13.7 cm^{-1}. Compared with previously reported FWHM values,[3-5] our h-BN fabricated by AP H_2 annealing shows high crystallinity.

Supplementary Fig. S3 (b) shows the FT-IR data of h-BN. Here, we found strong absorption peaks at

1365.4 cm^{-1} and 817.7 cm^{-1}. Those peaks were due to the in-plane and out-of-plane lattice vibrations, respectively.[6] These results corresponded to the reported data of h-BN.[7] The XPS spectrum, shown in Supplementary Fig. S3 (b), was introduced to analyse the elemental composition of our h-BN samples. The boron and nitrogen peak in Supplementary Fig. S3 (c) and (d) were located at 190.5 eV (boron 1s) and 398.2 eV (nitrogen 1s), which corresponds to the B and N peak in the XPS data of the previous reports.[8,9] Thus, we found that our sample consisted of sp^2 bonded materials.

4) TEM, SAED, and EELS analysis of h-BN

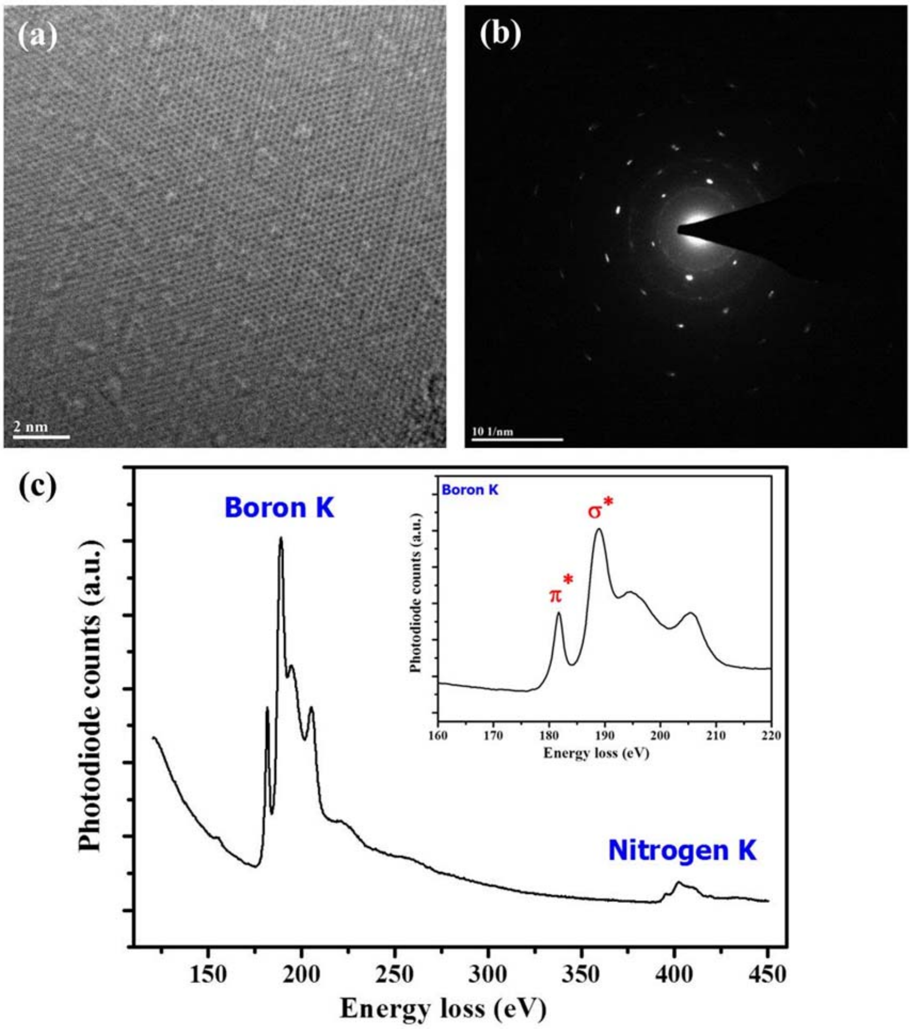

Figure S4. TEM, SAED, EELS data of h-BN (a) High resolution TEM image (b) SAED image, (c) EELS image, inset image of boron K-shell showed the characteristics of the sp^2 hybridised atoms of h-BN.

The high-resolution TEM images of the h-BN are shown in Supplementary Fig. S4 (a). The SAED data clearly showed a hexagonal diffraction pattern in Supplementary Fig. S4 (b). These indicate that the h-BN has high crystallinity. Electron energy loss spectroscopy (EELS) analysis and a EELS spectrum of h-BN was presented in Supplementary Fig. S4 (c) for the elemental analysis of the h-BN. The visible peaks corresponded to distinctive K-shell ionisation edges of boron and nitrogen, respectively.[10]

5) EBSD analysis of h-BN on the Ni foil with ECP/AP-10

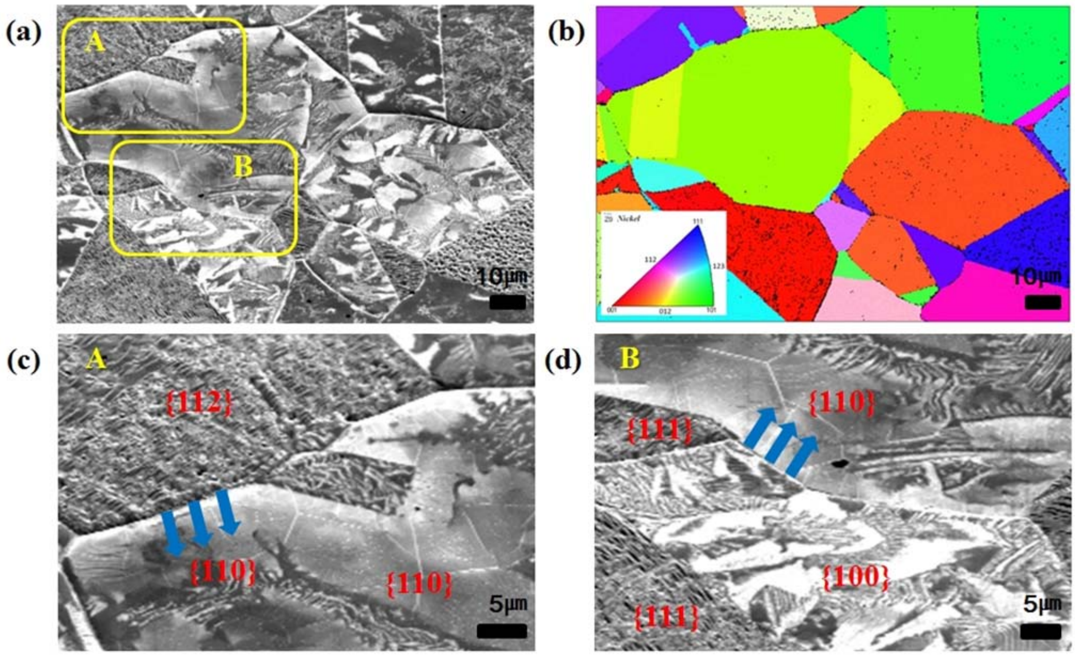

Figure S5. SEM images and EBSD analysis data of h-BN on the Ni foil with ECP/APH$_2$-10min; (a) SEM image (b) EBSD orientation map (c) SEM image of area A (d) SEM image of area B

6) The analysis of h-BN growth in various grain boundaries by using (a) EBSD, (b) SEM and (c) AES of the Ni foil with ECP/APH$_2$-10min.

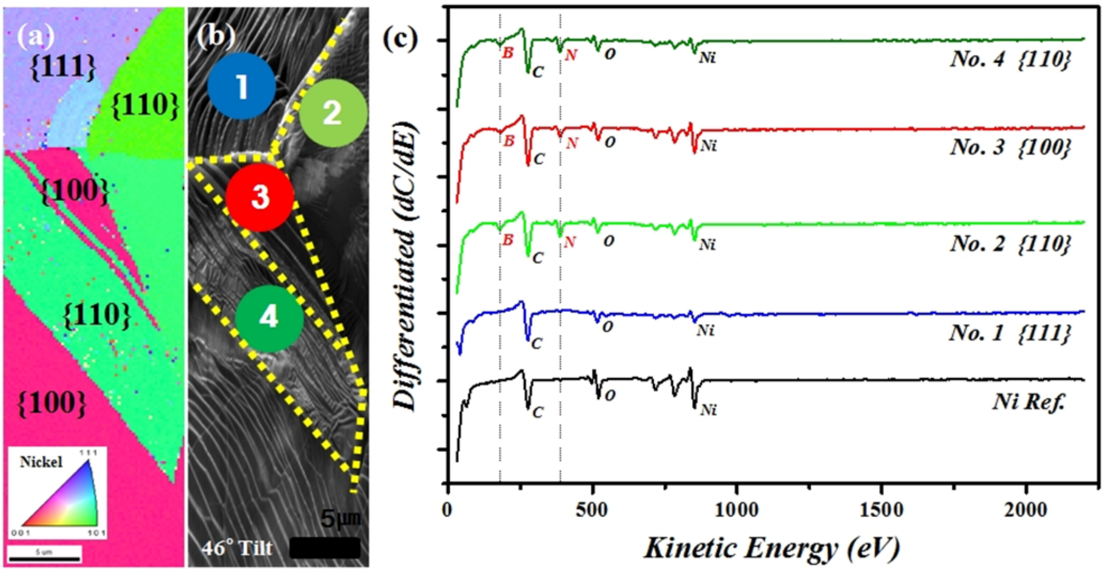

Figure S6. The analysis of h-BN growth in various grain boundaries by using (a) EBSD, (b) SEM and (c) AES of the Ni foil with ECP/APH$_2$-10min.

7. The comparison among RMS value, nucleation density and growth rate.

Table S1. The comparison among RMS value, nucleation density and growth rate.

	ECP/LPH$_2$-10min	ECP/APH$_2$-10min
RMS	3.142 nm	1.037 nm
Nucleation density	387.5 nuclei mm^{-2}	23.75 nuclei mm^{-2}
Growth rate	16.5 μm^2 min^{-2}	30.47 μm^2 min^{-2}

References

1. Cho, H et al., Parametric Study of Methanol Chemical Vapor Deposition Growth for Graphene. *Carbon letters* **13**, 205-211 (2012).

2. Gorbachev, R. V. et al. Hunting for monolayer boron nitride: optical and Raman signatures. *Small* **7**, 465-468 (2011).

3. Kubota, Y., Watanabe, K., Tsuda, O. & Taniguchi, T. Deep ultraviolet light-emitting hexagonal boron nitride synthesized at atmospheric pressure. *Science* **317**, 932-934 (2007).

4. Song, L. et al. Large scale growth and characterization of atomic hexagonal boron nitride layers. *Nano. Lett.* **10**, 3209-3215 (2010).

5. Lee, K. H. et al. Large-scale synthesis of high-quality hexagonal boron nitride nanosheets for large-area graphene electronics. Nano. Lett. **12**, 714-718 (2012).

6. Geick, R., Perry, C. & Rupprecht, G. Normal modes in hexagonal boron nitride. *Phys. Rev.* **146**, 543 (1966).

7. Shi, Y. et al. Synthesis of few-layer hexagonal boron nitride thin film by chemical vapor deposition. *Nano. Lett.* **10**, 4134-4139 (2010).

8. Tian, J. et al. Effect of nitrogen ion implantation on the microstructural transformation of boron film. *Thin Solid Films* **401**, 106-110 (2001).

9. Ismach, A. et al. Toward the Controlled Synthesis of Hexagonal Boron Nitride Films. *ACS Nano* **6**, 6378-6385 (2012).

10. Chopra, N. G. et al. Boron nitride nanotubes. *Science* **269**, 966-967 (1995).
